# Neuroinflammation, AD, and Dementia

**DOI:** 10.4061/2010/974026

**Published:** 2010-09-07

**Authors:** Marcella Reale, Talma Brenner, Nigel H. Greig, Nibaldo Inestrosa, Diana Paleacu

**Affiliations:** ^1^Department of Oncology and Neurosciences, ‘G. D'Annunzio' University of Chieti-Pescara, Italy; ^2^Department of Neurology, Hadassah Hebrew University Medical Center, P.O. Box 12000, Jerusalem 91120, Israel; ^3^Laboratory of Neurosciences, National Institute on Aging, Baltimore, MD 21224-6825, USA; ^4^Centro de Envejecimiento y Regeneración, Centro de Regulación Celular y Patología “Joaquín V. Luco,” Instituto Milenio, Facultad de Ciencias Biológicas, Pontificia Universidad Católica de Chile, Alameda 340, P.O. Box 8331150, Santiago, Chile; ^5^Neurology Service and Memory Clinic, Abarbanel Mental Health Center, 15 Keren Kayemet, Bat Yam 59110, Israel

This special issue of the *International Journal of Alzheimer's disease* contains a series of cuttingedge articles that deal with the broad issue of inflammatory and immunological disease mechanisms of Alzheimer disease (AD). Of necessity, these articles focus on selected topics but the mixture of contributions on signaling pathways, mediators, and articles addressing cell lines provides an overview and an insight into current areas of debate in inflammation and neurodegeneration. In particular, they highlight current interest in the interface between innate and adaptive immunity and present intriguing prospects for future therapeutic developments in neuroinflammation areas.

Immunohistochemical and molecular biological evidence accumulated over the past two decades has shown that the brain is capable of sustaining an innate immune response and that the result may be damaging to host cells. Neuroinflammation is a characteristic feature of both acute and chronic CNS disorders and is a process that results primarily from the presence of chronically activated glial cells (astrocytes and microglia) in the brain, and is a common feature of several neurodegenerative conditions. The evidence for a chronic inflammatory reaction in the brain is particularly strong in AD where it has been extensively studied. Mounting evidence indicates that microglial activation contributes to neuronal damage in neurodegenerative diseases, but beneficial aspects have also been identified. The concept that neuroinflammation is detrimental implies that glial cell activation precedes and causes neuronal degeneration, a sequence of events that appears to be at odds with experimental models of neurodegeneration in which glial cell activation occurs secondary to neuronal damage. There is abundant evidence that numerous substances involved in the promotion of inflammatory processes are present in the CNS of patients with such neurodegenerative diseases. Inflammation in the brain is silent because the brain does not possess pain fibers. It depends upon synthesis of inflammatory components by local neurons and glia, and especially resident phagocytes which, in the brain, are the microglia. Microglia, inflammatory cytokines, and the complement system appear to play significant roles. 

Dementia is a decline of cognitive functions (reasoning, memory, and other mental abilities). This decline eventually impairs the ability to carry out mundane activities such as driving; household chores, and personal care such as bathing, dressing, and feeding (often denoted as *activities of daily living*), or ADL. 

Proinflammatory mediators released by activated glial cells during brain inflammation have been proposed to contribute to neuropathology underlying cognitive deficits. Three major pathologies characterize the disease: senile plaques, neurofibrillary tangles, and inflammation. What distinguishes AD from other neurodegenerative diseases is the conspicuous presence of extracellular amyloid deposits in senile plaques, dystrophic neurite growth, and excessive tau phosphorylation. Senile plaques in AD brain are present in different stages of maturity, ranging from diffuse to neuritic to dense core, but they all contain amyloid beta protein (A*β*). A*β* is a peptide that forms insoluble and pathological extracellular aggregates that attract microglial cells, as suggested by microglia clustering at A*β* deposition sites.

Duleu et al., using an improved ELISA procedure, observed in AD patient sera, circulating IgA isotype antibodies directed against tryptophan metabolites. The paper confirms that neurotoxic tryptophan metabolites are implicated in neurodegenerative diseases. The activation of the IDO pathway leads to an over expression of tryptophan metabolites, which are implicated in neuroinflammation in AD. The identification of circulating antibodies directed against IDO/THO pathway metabolites elucidates the etiology of AD. 

In the paper entitled “*Microglial immunoreceptor tyrosine-based activation and inhibition motif signaling in neuroinflammation,*” Linnartz et al. reviewed the involvement of ITAM- and ITIMsignaling receptors in the central nervous system (CNS) innate immune responses and neuroinflammation as modulator of microglial phagocytosis and cytokine expression. In the mammalian CNS, essential molecules in this process are Fc receptors and DAP12-associated receptors which trigger the microglial immunoreceptor tyrosine-based activation motif (ITAM)-Syk-signaling cascade. The authors indicated how ITAM- and ITIM-signaling receptors modulate microglial phagocytosis and cytokine expression during neuroinflammatory processes. They also described how their dysfunction could lead to impaired phagocytic clearance and neurodegeneration triggered by chronic inflammation. 

Hjorth et al. using human CHME3 microglia showed intracellular A*β*_1–42_ colocalized with lysosome-associated membrane protein-2. This indicated that phagocytosis was increased by interferon-*γ*, and to a lesser degree, by Protollin, a proteasome-based adjuvant. Phagocytosis of A*β*_1–42_ is associated with the expression of inflammatory markers. A*β*_1–42_ and interferon-*γ* decreased BDNF secretion suggesting a new neuropathological role for A*β*_1–42_ and inflammation accompanying AD.

In another review, Dyall described neuroprotective effects of eicosapentaenoic acid (EPA) and docosahexaenoic acid (DHA) in AD. Inflammation and oxidative stress appear to be key features contributing to AD, and omega-3 polyunsaturated fats, EPA, and DHA have wellcharacterised effects on inflammation and may have neuroprotective effects in a number of neurodegenerative conditions. The role of EPA and DHA in modulating oxidative stress and inflammation and their potential as therapeutic agents were emphasized. 

In the paper entitled “*Interleukin-10 promoter polymorphism in mild cognitive impairment and in its clinical evolution*,” Arosio et al. strengthen the theory that the overall risk of developing AD may be governed by a multifactorial “susceptibility profile” and that polymorphisms of cytokine genes can affect neurodegeneration and its clinical progression. In their study, the authors have analysed genotype and allele frequencies of A allele of −1082 polymorphism (G/A) of interleukin-10 (IL-10) in 138 subjects with mild cognitive impairment (MCI) diagnosed respectively as amnestic (a-MCI) and multiple impaired cognitive domains (mcd-MCI). The homozygosis for the A allele of this polymorphism of IL-10 promotes an higher risk of AD and reduced IL-10 generation in peripheral cells after amyloid stimulation. Thus, the authors proposed that IL-10 may partly explain the conversion of a-MCI to AD or, at least, be a genetic marker of susceptibility.

The final paper of this special issue by Krause et al. reviews the detrimental and beneficial role of neuroinflammation in AD. The authors focus on controversies in the field of microglia activation, attempting to shed light on whether neuroinflammation is associated with brain tissue damage and functional impairment or whether damage-limiting activity occurs. The possible limitations, options and advantages of anti-inflammatory treatment are discussed and possible implications resulting from neuroinflammation for AD therapy. 

*Marcella Reale* *Marcella Reale* *Talma Brenner* *Talma Brenner* *Nigel H. Greig* *Nigel H. Greig* *Nibaldo Inestrosa* *Nibaldo Inestrosa* *Diana Paleacu* *Diana Paleacu*

